# A Rise in Aspartate Transaminase and Alanine Transaminase Associated With Ondansetron Administration in a Pregnant Female

**DOI:** 10.7759/cureus.11540

**Published:** 2020-11-18

**Authors:** Michael Couse, Saba Syed

**Affiliations:** 1 Psychiatry, Olive View - University of California, Los Angeles (UCLA) Medical Center, Los Angeles, USA

**Keywords:** toxicology, pharmacology, liver damage

## Abstract

Ondansetron (ODSN) is a commonly used and well-tolerated anti-emetic used during pregnancy. Upon review, three cases were identified in which liver function tests (LFTs) were elevated after ODSN administration without concurrent chemotherapy. In this report, we present a case of a 28-year-old pregnant female experiencing psychosis who developed serum level elevation of aspartate transaminase (AST) and alanine transaminase (ALT). An extensive workup left drug-induced liver injury as the most likely etiology. On retrospective review, we were able to demonstrate a clear relationship between ODSN administration and the rise of ALT and AST serum levels.

A 28-year-old female gravida 3 para 2, who was 28 weeks pregnant, presented to a county hospital with new-onset psychotic features and persistent vomiting. She was diagnosed with major depressive disorder (MDD) with psychotic features and hyperemesis gravidarum (HG). She was started on medications to treat both conditions including as needed ODSN. Several days after admission, her serum levels of AST and ALT began to rise, eventually plateauing at an AST value of 267 units/liter (U/L) and an ALT value of 605 U/L on hospital day 10 and subsequently started decreasing. A comprehensive workup was performed to identify the cause of her hypertransaminasemia. Multiple causes were ruled out, with the most likely etiology determined to be drug-induced liver injury. She was admitted to the psychiatric emergency room (PER) on two separate occasions due to psychotic decompensation from medication noncompliance. On both occasions, her AST and ALT values were within normal limits. Ten weeks from the first admission she came for delivery. The patient had been medication compliant prior to delivery and displayed no features of psychosis. Notably, her AST and ALT were elevated and continued to rise until her delivery after which the serum levels decreased.

A sharp rise in the plasma concentration of ALT and AST indicates an acute injury liver. As noted in the case presentation, medical workup ruled out many possible etiologies of her hypertransaminasemia. An important differential to consider was HG, however, based on the history and timing of LFT rise, this differential was unlikely. With other differential diagnoses ruled out, medication-induced hepatotoxicity was the most likely diagnosis. Next, by reviewing the medication administration record we found a temporal relationship between the onset and discontinuation of ODSN and the pattern of AST and ALT levels. Temporal relationships between AST and ALT levels were able to be excluded from all other medications this patient received.

With limited reports indicating the hepatotoxicity potential of ODSN in the absence of concomitant chemotherapy administration, we hope this report adds to the literature and potentially assists future clinical decision making.

## Introduction

Ondansetron (ODSN) is a commonly used anti-emetic during pregnancy. The Royal College of Obstetricians and Gynecologists and the American College of Obstetricians and Gynecologists both include ODSN as a second-line agent for the management of nausea and vomiting in pregnancy [1-2]. According to the package insert, in patients receiving both chemotherapy and ODSN, 1%-2% demonstrated an aspartate transaminase (AST) or alanine transaminase (ALT) elevation greater than two times the normal range, however, there is no further reference of higher elevations or elevations seen without concomitant chemotherapy administration [3]. LiverTox is a National Institutes of Health supported, up-to-date “living textbook” on liver injury attributable to medications [4-5]. It acts as a central repository of information with a comprehensive bibliography of references prepared principally from searches of PubMed and textbooks on hepatotoxicity. Upon review of the references in the “Serotonin 5-HT3 Receptor Antagonists” section, three cases were identified in which liver function tests (LFTs) were elevated after ODSN administration without concurrent chemotherapy [6-8]. In this report, we present a case of a 28-year-old pregnant female experiencing psychosis who develops AST and ALT elevations following ODSN administration. Although the patient has multiple risk factors for LFT elevation and was administered numerous medications, ODSN becomes the clear causal agent upon careful investigation. Based on our review, we believe this is the first reported case of ODSN-induced hepatotoxicity during pregnancy.

## Case presentation

A 28-year-old female gravida 3 para 2, who was 28 weeks pregnant, presented to a county hospital with complaints of auditory hallucinations (AH), visual hallucinations (VH), and paranoia. The patient had no known psychiatric history with the onset of psychotic symptoms beginning one week prior to presentation. It was later confirmed by collateral contact that the patient was suffering from three months of depressed mood, anhedonia, crying spells, decreased sleep, energy, and appetite. On presentation, the physical exam was benign, vitals, complete blood count (CBC), comprehensive metabolic panel (CMP) were within normal limits (WNL). The patient was not taking medications, herbal supplements, or illicit drugs and urine drug screen (UDS) was negative. CT and noncontrast MRI were both unremarkable. The patient was diagnosed with major depressive disorder (MDD), severe with psychotic features, and was started on sertraline (SRT) and olanzapine (OLZ). SRT was titrated up to 50 mg per day and OLZ to 20 mg per day.

Concurrently, the patient admitted to four weeks of nausea, vomiting, and limited oral intake resulting in a 10-pound weight loss. She was diagnosed with hyperemesis gravidarum (HG) and started on the combination of IV famotidine 20 mg twice a day (BID), doxylamine succinate 12.5 mg four times per day (QID), and pyridoxine 25 mg QID. Despite this treatment, her nausea and vomiting continued resulting in the addition of ODSN to be utilized on an as-needed basis (PRN). The first administration of ODSN was one day after admission with the majority of doses given three to eight days after admission. The total daily doses never exceeded 12 mg.

Five days following admission, LFTs were assessed with the AST measuring at 73 units/liter (U/L) and ALT at 113 U/L. On the next day, the AST and ALT continued to rise to 111 and 176 U/L respectively. A comprehensive workup was performed to identify the cause of her hypertransaminasemia. Laboratory tests assessing for hepatitis A, B, and C were all negative, anti-smooth muscle antibody was negative and antinuclear antibodies, alpha-1 antitrypsin, ceruloplasmin, total bilirubin, and alkaline phosphatase levels were all normal. The right upper quadrant ultrasound showed no abnormalities. Pre-eclampsia and the associated hemolysis, elevated liver enzymes, and a low platelet count (HELLP) syndrome were ruled out due to normal measurements of blood pressure, urine protein to creatinine ratio via 24-hour urine collection, platelet count, and hemoglobin level. With most causes ruled out, drug-induced liver injury became the leading explanation for her elevated transaminases. As such, her medication regimen was reviewed and OLZ was identified as a possible causal agent due to the known association of OLZ and hepatotoxicity [4]. The patient was switched to haloperidol (HDL) and titrated up to a total daily dosage of 20 mg. Despite this switch, her LFTs continued to rise. Eight days after her admission, the patient’s nausea and vomiting improved, resulting in tolerance of oral intake and requiring only two doses of 4 mg ODSN for the remaining six days of her hospitalization. Some 10 days following her admission, her transaminases plateaued with AST measured at 267 U/L and ALT at 605 U/L. Her transaminases down trended for the remainder of her hospitalization approaching normal limits by the time of discharge. With her psychosis and nausea significantly improved and transaminases approaching normal range, she was discharged after 14 days in the hospital. She was instructed to take HDL 20 mg per day, SRT 50 mg per day, famotidine 20 mg BID, pyridoxine 25 mg QID, ODSN 4 mg PRN, and doxylamine 12.5 mg PRN.

In the following month, the patient was admitted on two additional instances to the psychiatric emergency room (PER) for psychosis prior to delivery. On both occasions, it was determined, with the assistance of collateral information that her decompensation was due to medication noncompliance. On the first admission, her CBC and CMP were WNL and UDS was negative. She quickly reconstituted when she was restarted on HDL and was discharged home in two days. Some 19 days following this discharge, the patient presented resulting in her third admission. Similar to her previous encounter, no abnormalities were found on laboratory testing or UDS and she quickly reconstituted once she restarted HDL. After carefully weighing the risks and benefits, it was determined she would benefit from a long-acting injectable form of HDL, Haldol Decanoate. The patient received a 100 mg intramuscular injection of Haldol Decanoate, continued on oral HDL and SRT, and discharged home.

Seventy-two days following her initial admission, the patient presented for delivery. Upon admission, the patient was linear, organized, and showed no signs of psychosis. She claimed to be adherent to HDL 10 mg, SRT 50 mg, and utilized ODSN 4 mg PRN. She denied the continuation of famotidine, pyridoxine, and doxylamine succinate. It is unclear how frequently she self-administered ODSN. On admission, the patient was nauseous with a low appetite. Her AST and ALT were measured at 348 and 575 U/L, respectively. The patient received a dose of 4 mg ODSN on the first day of admission and was continued on SRT and HDL. She received another dose of 4 mg ODSN on her third day of hospitalization and her transaminases continued to rise. Later that night, she delivered via planned cesarean section. Following her delivery, she experienced a rapid resolution of her nausea and did not receive additional ODSN. Over the next three days, she recovered from her delivery and her transaminases significantly down trended resulting in discharge.

## Discussion

The AST and ALT are the most specific indicators of hepatocellular necrosis and a sharp rise of their plasma concentration indicates acute injury liver. As noted in the case presentation, medical workup ruled out many possible etiologies of her hypertransaminasemia including cirrhosis, autoimmune and viral hepatitis, cholestasis, alpha-1 antitrypsin deficiency, Wilson’s disease, pre-eclampsia, HELLP syndrome, and acute fatty liver of pregnancy. Instances of elevated LFTs have been described in HG and without a diagnostic test to implicate HG, it cannot be completely ruled out [9]. However, based on history, this differential is unlikely as the patient was experiencing nausea and vomiting for weeks leading up to her first presentation yet her LFTs were WNL. With other differential diagnoses ruled out, medication-induced hepatotoxicity is the most likely diagnosis.

**Figure 1:**
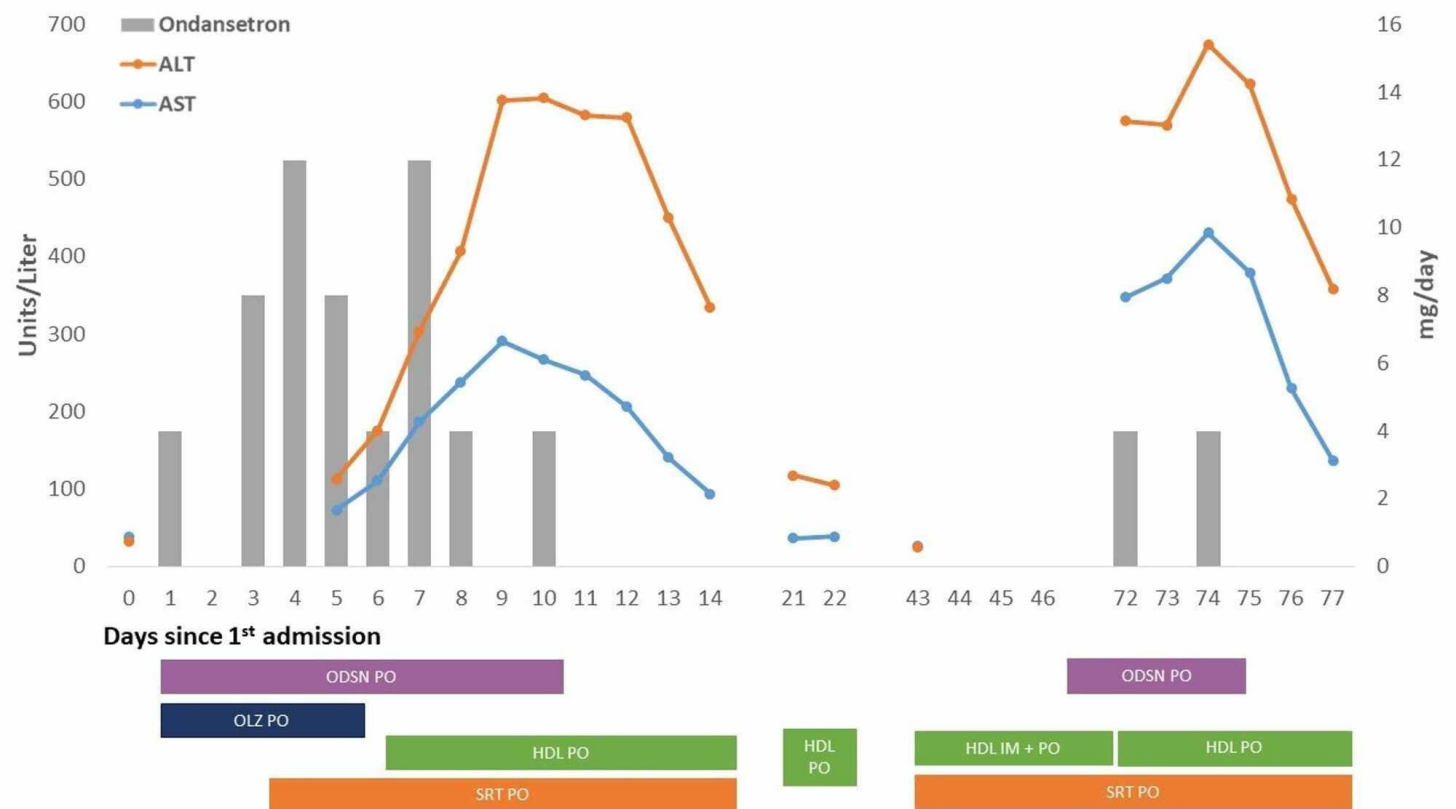
AST and ALT serum levels plotted with ODSN dosage and duration of medication administration. Days following original presentation are plotted on the X-axis with “Day 0” corresponding to the day of first presentation to the hospital. Serum levels of AST and ALT are plotted on the Y-axis with the total daily dosage of ODSN administered while hospitalized on the secondary Y-axis. Shaded rectangles beneath the X-axis indicate the days a medication was consumed including during periods when the patient was not hospitalized. AST, aspartate transaminase; ALT, alanine transaminase; ODSN, ondansetron; OLZ, olanzapine; HDL, haloperidol; SRT, sertraline

Next, we attempted to find whether a temporal relationship exists between the onset and discontinuation of various medications and the pattern of AST and ALT levels (Figure 1).

Recognizing that the half-life of AST and ALT is 17 and 47 hours respectively, the offending agent would need to result in continued hepatotoxicity to observe the approximately 96-hour rise of transaminases that occurred between days five and nine after admission [10]. Similarly, the offending agent would have to result in little to no hepatotoxicity during the decline of transaminases observed from days 11 to 14. Of the medications administered to the patient, HDL, OLZ, ODSN, SRT, famotidine, doxylamine, and pyridoxine were administered prior to or at the initial rise of transaminases. Further, only OLZ and ODSN were discontinued or significantly reduced prior to the plateau and decline of transaminases. Preceding her second and third presentations, the patient was noncompliant with any of her medications which are in accordance with her presentation of decompensated psychosis. Additionally, when not taking ODSN, but continued with HDL and SRT, her transaminase levels were WNL on both presentations. To prevent additional relapses, she received a long-acting antipsychotic injection, which according to both the patient and collateral, resulted in continued psychosis remission leading up to her fourth admission for delivery. The patient reports ODSN usage during this time and subsequently presents with elevated transaminases. Upon admission, the patient received two more doses of ODSN resulting in continued elevation of her transaminases. Similar to her original presentation, following cessation of ODSN treatment, a swift decline in transaminases occurred.

## Conclusions

In this report, we describe the case of a pregnant female presenting to a county hospital due to the onset of MDD with psychosis and HG. Multiple days after admission, her transaminases begin to significantly rise resulting in a thorough workup to elucidate the etiology. Based on history, physical exam, laboratory tests, and imaging studies, many causes of elevated transaminases were ruled out leaving medication-induced hepatotoxicity the most likely diagnosis. After careful review of all medications administered, we identified a clear temporal relationship between ODSN dosing and transaminase levels. Based on our review of the literature, we believe this is one of the few cases reported of ODSN-induced hepatotoxicity and the first identified during pregnancy.

In 2017, ODSN was the 31st most commonly prescribed medication in the United States and yet only a small number of reports are published implicating hepatotoxicity indicating its tolerability. Regardless, all medications have the potential to result in hepatotoxicity, and when medication-induced hepatotoxicity is suspected all recently administrated medications should be reviewed. In a clinically complex situation like the case presented above, polypharmacy is a near necessity, and therefore determining the causal agent is difficult. Often, clues in the history can aid in determining which agent to discontinue but, at times, the only approach is to rely on published reports and apply past experiences with a current clinical case. With limited reports indicating the hepatotoxicity potential of ODSN in the absence of concomitant chemotherapy administration, we hope this report adds to the literature and potentially assist future clinical decision making.
